# Is dietary zinc protective for type 2 diabetes? Results from the Australian longitudinal study on women’s health

**DOI:** 10.1186/1472-6823-13-40

**Published:** 2013-10-04

**Authors:** Khanrin Phungamla Vashum, Mark McEvoy, Zumin Shi, Abul Hasnat Milton, Md Rafiqul Islam, David Sibbritt, Amanda Patterson, Julie Byles, Deborah Loxton, John Attia

**Affiliations:** 1 Centre for Clinical Epidemiology & Biostatistics, School of Medicine & Public Health, University of Newcastle, HMRI Building, Callaghan-2308 Newcastle, NSW, Australia; 2 Discipline of Medicine, University of Adelaide, Adelaide, South Australia; 3 School of Health Sciences, University of Newcastle, Newcastle, Australia; 4 Research Centre for Gender, Health and Ageing, University of Newcastle, Newcastle, New South Wales, Australia; 5 Hunter Medical Research Institute, and Department of General Medicine, John Hunter Hospital, Newcastle, Australia

**Keywords:** Diabetes, Australia, Women & Zinc

## Abstract

**Background:**

Animal studies have shown that zinc intake has protective effects against type 2 diabetes, but few studies have been conducted to examine this relationship in humans. The aim of this study is to investigate if dietary zinc is associated with risk of type 2 diabetes in a longitudinal study of mid-age Australian women.

**Methods:**

Data were collected from a cohort of women aged 45-50 years at baseline, participating in the Australian Longitudinal Study on Women’s Health. A validated food frequency questionnaire was used to assess dietary intake and other nutrients. Predictors of 6-year incidence of type 2 diabetes were examined using multivariable logistic regression.

**Results:**

From 8921 participants, 333 incident cases of type 2 diabetes were identified over 6 years of follow-up. After adjustment for dietary and non-dietary factors, the highest quintile dietary zinc intake had almost half the odds of developing type 2 diabetes (OR = 0.50, 95% C.I. 0.32–0.77) compared with the lowest quintile. Similar findings were observed for the zinc/iron ratio; the highest quintile had half the odds of developing type 2 diabetes (OR = 0.50, 95% C.I 0.30-0.83) after multivariable adjustment of covariates.

**Conclusions:**

Higher total dietary zinc intake and high zinc/iron ratio are associated with lower risk of type 2 diabetes in women. This finding is a positive step towards further research to determine if zinc supplementation may reduce the risk of developing type 2 diabetes.

## Background

Diabetes, a disorder of metabolism results in substantial morbidity and mortality, primarily from macrovascular (myocardial infarction, stroke, and peripheral vascular disease), and microvascular effects (retinopathy, nephropathy, and neuropathy). The World Health Organization (WHO) estimated that 3.4 million people worldwide died from consequences of high fasting blood sugar in 2010 [1]. Currently 347 million adults worldwide have diabetes; the prevalence is continuing to rise and is expected to be the 7th leading cause of death by 2030 [1]. This will have the greatest impact on the productive population of countries, as people under the age of 70 years constitute almost half of all diabetic deaths.

Molecular and cellular studies have demonstrated that the mineral zinc plays a key role in the synthesis and action of insulin under normal physiological conditions and in type 2 diabetes (T2D). Observational studies have reported an association between reduced serum zinc and established T2D [2]. Protective effects of zinc supplementation have also been demonstrated in rodent models of T2D [3], but this has not been properly tested in humans.

Epidemiological studies have observed an association between reduced zinc status and T2D [4,5]. This association may simply be due to loss of zinc through the kidneys due to diabetic nephropathy; indeed, a number of studies have shown that urinary excretion of zinc is increased and serum levels decreased in T2D patients compared with controls [5]. A study by Marreiro et al found that 35.5% of study subjects with T2D had higher urinary zinc excretion than normal [6]. However, other lines of evidence support a more direct causative role of zinc in the pathogenesis of T2D. A few studies have examined the effect of zinc supplementation in T2D patients with many showing an improvement in glycaemic control [7-9]. Only one study has examined the use of zinc supplementation for the primary prevention of T2D [6]. This study was identified and the only study included in a recent Cochrane review that examined the role of zinc supplementation for the primary prevention of T2D and found that there was insufficient evidence to suggest the use of zinc supplementation in the prevention of T2D [10]. Recent support for the use of zinc supplementation for reducing the risk of T2D comes from the Nurses’ Health Study (n ~ 82,000) where higher dietary zinc intake was associated with a lower risk of developing T2D in women [11]. However, another study in a Chinese population did not find an association between dietary zinc intake and hyperglycaemia but did find an association with the dietary zinc to haeme iron ratio [12]. In light of these findings we have used a large population-based cohort of women to examine these associations in an Australian context. In addition, this study also examines dietary zinc to iron ratio as iron is known to interact with the absorption of zinc and a recent study showed that zinc to haeme iron ratio was inversely associated with risk of diabetes in US women [11].

## Methods

### The Australian longitudinal study on women’s health

The Australian Longitudinal Study on Women’s Health (ALSWH) is a prospective study examining the health and wellbeing of three cohorts of women aged 18–23 years (young), 45–50 years (mid-aged), and 70–75 years (older) at the time of the initial surveys in 1996. Women were selected randomly within each age group from the National Medicare Health Insurance Database (which includes all permanent residents of Australia regardless of age, including immigrants and refugees) with intentional overrepresentation of women living in rural and remote areas, which was achieved by sampling women in these areas at twice the rate of women in urban areas. Further details of the recruitment methods and response rates have been described elsewhere [13]. The study collects self-reported data using mailed surveys at 2- to 3-year intervals from 40,000 women living in all states and territories of Australia. The surveys include questions about health conditions, symptoms, and diagnoses; use of health services; health-related quality of life; social circumstances, including work and time use; demographic factors; and health behaviours. Complete details of each survey (S) are on the study website (available at http://www.alswh.org.au). Informed written consent was obtained from all participants in 1996, with ethical clearance for the study obtained from the University of Newcastle. Ethics committees at the Universities of Newcastle and Queensland approved the ALSWH. This article only includes data from the mid-aged cohort as this age group (45-50 years) were at higher risk of developing diabetes than the other two cohorts (18–23 and 70–75 years) of women and were the only age group with food frequency questionnaire (FFQ) data collected. There were five waves of data collection from 1996 to 2007 (S1, 1996; S2, 1998; S3, 2001; S4, 2004; and S5, 2007). Surveys 1, 2 and 3 consisted of 13716, 12338, and 11228 women respectively. The response rate for S3 of the mid age cohort was 83% of women who had completed S1 and had not died (n-115) or become too ill to complete further surveys (n = 21). Other non-respondents included those who were unable to be contacted (n = 930), were contacted but did not complete the survey (n = 998), and those who withdrew from the project (n = 242) [13]. Of the women who completed S3 (then aged 50–55 years), 11196 completed the food frequency questionnaire (FFQ) and 8921 participants remained in the study and were available for analysis by S5. Those who were lost to follow-up were not significantly different but were more likely to be born outside Australia, less educated, or a current smoker.

### Dietary assessment

At S3, dietary intake was assessed using an FFQ known as the Dietary Questionnaire for Epidemiological Studies (DQES) Version 2. Both the development of the questionnaire [14] and its validation in mid-aged Australian women has been previously reported [15]. This questionnaire asks respondents to report their usual consumption of 74 foods and six alcoholic beverages over the preceding 12 months using a 10-point frequency scale. Additional questions are asked about the number of serves or type of fruit, vegetables, bread, dairy products, eggs, fat spreads and sugar and further details are provided in Hodge et al. [15]. Nutrient intakes were computed from NUTTAB 1995, a national government food composition database of Australian foods NUTTAB95 [16], using software developed by the Cancer Council of Victoria. The validation of the FFQ against a 7-day weighted food record showed Pearson correlation coefficient = 0.4 for dietary zinc intake. The FFQ validation study deemed the correlation coefficient acceptable as it is of similar magnitude to those previously reported [15].

### Ascertainment of type 2 diabetes

At each survey women were asked if a doctor had told them that they had T2D. At S1 they were asked if they had ever had a diagnosis of T2D. At S2, S3, S4, and S5 they were asked whether they had been diagnosed with T2D in the time period that had elapsed since the previous survey. Prevalence of T2D at each survey was defined as the proportion of these women who reported at that survey or a previous survey that they had been told they had T2D.

### Measurement of non-dietary factors

Social and behavioural factors were based on information collected at S3 (AIHW 2001). Participants were asked to report frequency of engaging in vigorous (e.g., aerobics, jogging) and less vigorous (e.g., walking and swimming) exercise lasting for <20 min in a normal week. Responses were scored using approximate weekly frequencies of exercise (never = 0, once a week = 1, 2 or 3 times per week = 2.5, 4–6 times per week =5, every day = 7, and more than once a day = 10) and then weighted to reflect the intensity of the activity (vigorous = 5 and less vigorous = 3). The resulting physical activity scores ranged from 0 to 80 and were categorized as “nil/sedentary (<5),” “low (5 to 15),” “moderate (16 to 25),” or “high (>25).” A score of 15 is commensurate with the current recommendation of moderate intensity activity on most days of the week. This measure is described in more detail elsewhere [17] and has previously been shown to have acceptable test-retest reliability [18]. Standard questions were used to categorize respondents as never-smoker, ex-smoker, or current smoker; the latter was grouped as smoking less than 10 cigarettes (c) per day (<10c/d), 10-19 c/d and > =20 c/d.

Body mass index (BMI) was calculated as self-reported weight (kg) divided by the square of estimated height (m^2^). Medical history of arthritis, congestive heart disease (CHD), stroke, hypertension (HT), asthma and depression along with use of hormone replacement therapy (HRT) (coded as either yes or no) were all self-reported. The participants were also asked to report the number of supplements being used and categorised as taking multivitamin & mineral supplements (yes or no).

### Statistical analysis

Chi square test was used to compare differences in categorical variables, and ANOVA was used to compare mean values of continuous variables between groups. Predictors of 6-year incidence of T2D were examined using forward stepwise multivariable logistic regression in stepped approach, with the main predictor being energy adjusted zinc and zinc/iron ratio measured at S3 used to predict incidence of T2D by S5. Women who first reported a diagnosis of T2D at S1, S2, or S3 (prevalent cases) were excluded from this analysis (*N = 567*). The macronutrient variables are adjusted for total energy intake by calculating their component of total energy (as a%). The micronutrients were adjusted for total energy by regressing (using linear regression) the natural log of the micronutrient on the natural log of total energy and extracting the standardized residuals.

The multivariable analysis controlled for dietary factors (energy adjusted fibre, fat and iron) and non-dietary factors (BMI, smoking status, HRT, exercise group, history (yes/no) of arthritis, CHD, HT, asthma and depression). P values for trends were conducted by treating quintile of energy-adjusted zinc as a continuous variable. Statistical significance was considered when 2 sided p < 0.05. STATA software version 11 was used for all statistical analyses.

## Results

At the end of the 6 years follow-up 333 incident cases of T2D were identified out of 8,921 participants. Table 1 describes characteristics of participants at survey 3 by quintile of energy-adjusted zinc. Dietary zinc intake was divided into quintiles of energy-adjusted zinc with quintile 1 as the lowest intake and quintile 5 the highest intake. The dietary zinc intake of the lowest and highest quintile was 5.94 mg (95% CI 5.90 -5.99) and 17.35 mg (95% CI 17.12-17.59) respectively and the mean intake was 10.66 mg/day. At baseline (Table 1) non-dietary factors found to be significantly different across quintiles included smoking status, exercise, BMI, HRT, HT, asthma and depression. Energy-adjusted dietary factors found to be significantly different across quintiles includes carbohydrates, total protein, total fat (including cholesterol and saturated, polyunsaturated, monounsaturated fat), dietary fibre, minerals (iron, calcium, magnesium, sodium and potassium) and vitamins, which includes retinol, vitamin C and E. Those in the highest quintile of intake were more likely to smoke greater than 20 cigarettes per day and have a history of hypertension. Women in the lower quintiles were found to have a more sedentary life with nil or little exercise but interestingly those with higher intake of zinc also had higher BMI. Of particular interest in this investigation, those in the highest quintile of zinc intake also had the highest intake of dietary iron. The most commonly recorded dietary source of zinc was meat, fish and poultry as the major contributors, though cereals and dairy products were also a substantial source.

**Table 1:** Characteristics of subjects at survey 3 by quintile of energy-adjusted zinc

		**Quintile of energy-adjusted zinc**			
**Characteristic**	**Sub group/or mean(Std)**	**Q1 Lowest (n = 1785)**	**Q3 Middle (n = 1784)**	**Q5 Highest (n = 1784)**	**p-value**
Smoking status	Never smoker	948 (53%)	1033 (58%)	922 (52%)	<0.001
	Former smoker	568 (32%)	531 (30%)	569 (32%)	
	smoker < 10 c/d	84 (4.7%)	75 (4.2%)	76 (4.3%)	
	smoker 10-19 c/d	77 (4.3%)	64 (3.6%)	81 (4.6%)	
	smoker > = 20 c/d	100 (5.6%)	76 (4.3%)	130 (7.3%)	
Exercise group	Nil/sedentary	321 (19%)	271 (16%)	323 (19%)	0.003
	Low	621 (37%)	683 (40%)	622 (36%)	
	Moderate	318 (19%)	355 (21%)	338 (20%)	
	High	437 (26%)	408 (24%)	433 (25%)	
Hormone replacement therapy	No	1250 (70%)	1193 (67%)	1160 (65%)	0.020
	Yes	535 (30%)	591 (33%)	624 (35%)	
Heart disease	No	1743 (99%)	1741 (98%)	1735 (98%)	0.077
	Yes	26 (1.5%)	28 (1.6%)	30 (1.7%)	
Hypertension	No	1498 (85%)	1495 (85%)	1449 (82%)	0.038
	Yes	271 (15%)	274 (15%)	316 (18%)	
Arthritis	No	1409 (80%)	1358 (77%)	1375 (78%)	0.342
	Yes	360 (20%)	411 (23%)	390 (22%)	
Asthma	No	1568 (89%)	1602 (91%)	1584 (90%)	0.038
	Yes	201 (11%)	167 (9.4%)	181 (10%)	
Depression	No	1538 (87%)	1605 (91%)	1597 (90%)	0.001
	Yes	231 (13%)	164 (9.3%)	168 (9.5%)	
Age	mean (SD)	52.6 (1.4)	52.6 (1.5)	52.4 (1.5)	0.004
Body mass index	mean (SD)	26.0 (5.4)	26.5 (5.1)	27.2 (5.4)	<0.001
Total energy intake	mean (SD)	6676 (2415)	6604 (2275)	6687 (2790)	0.508
Alcohol intake	mean (SD)	6.2 (11.0)	8.3 (12.1)	9.7 (14.9)	<0.001
Number of supplements	mean (SD)	1.1 (1.1)	1.1 (1.1)	1.0 (1.1)	0.163
Carbohydrates (% of energy)	mean (SD)	48.2 (5.8)	45.6 (5.8)	41.0 (7.5)	<0.001
Dietary fibre (% of energy)	mean (SD)	2.4 (0.7)	2.5 (0.6)	2.5 (0.8)	<0.001
Total protein (% of energy)	mean (SD)	17.1 (2.1)	20.8 (1.7)	25.1 (2.8)	<0.001
Total fat (% of energy)	mean (SD)	35.5 (5.5)	34.3 (5.9)	34.5 (6.6)	<0.001
Saturated fat (energy adjusted)	mean (SD)	14.0 (3.8)	13.5 (3.4)	13.9 (3.3)	<0.001
Polyunsaturated fat (% of energy)	mean (SD)	6.3 (2.3)	5.5 (1.9)	4.7 (1.5)	<0.001
Monounsaturated fat (% of energy)	mean (SD)	12.1 (2.2)	12.1 (2.4)	12.6 (2.8)	<0.001
Iron (energy adjusted)	mean (SD)	−0.605 (0.984)	0.022 (0.905)	0.543 (0.897)	<0.001
Cholesterol (energy adjusted)	mean (SD)	−0.584 (1.170)	−0.043 (0.827)	0.607 (0.855)	<0.001
Retinol (energy adjusted)	mean (SD)	0.451 (0.947)	0.030 (0.932)	−0.525 (0.993)	<0.001
Vitamin C (energy adjusted)	mean (SD)	−0.092 (1.198)	0.024 (0.957)	0.028 (0.895)	0.001
Vitamin E (energy adjusted)	mean (SD)	0.346 (1.094)	0.035 (0.923)	−0.407 (0.939)	<0.001
Calcium (energy adjusted)	mean (SD)	−0.374 (0.901)	0.126 (0.940)	0.089 (1.166)	<0.001
Magnesium (energy adjusted)	mean (SD)	−0.453 (1.001)	0.064 (0.901)	0.268 (1.069)	<0.001
Sodium (energy adjusted)	mean (SD)	−0.432 (1.030)	0.026 (0.930)	0.345 (0.999)	<0.001
Potassium (energy adjusted)	mean (SD)	−0.594 (1.072)	0.067 (0.891)	0.454 (0.915)	<0.001

Table 2 shows the median value, along with the minimum and maximum values of energy-adjusted zinc for each quintile of dietary zinc. These values represent the standardized residuals after adjusting for total energy intake. For example, the median values of energy-adjusted zinc in Q1 and Q5 are -1.25 and 1.24 respectively indicating that the middle value for energy-adjusted zinc in Q5 is much higher than that of Q1. In an age adjusted analysis there was no significant association across quintiles between energy-adjusted zinc and risk of T2D (Table 2). After adjustment for non-dietary factors including age, BMI, smoking, HRT, exercise group, history of arthritis, CHD, HT, asthma and depression, an overall decrease in the odds of developing T2D across the quintiles was observed, which was statistically significant (p = 0.010). Further adjustment for dietary factors (energy adjusted fibre, iron and fat) strengthened the association (p = 0.004). Additional adjustment for alcohol intake and use of supplements (multivitamins or minerals) also showed a statistically significant decrease in the odds of developing T2D across quintiles (P_trend_ = 0.006). Compared with the lowest quintile of energy-adjusted zinc those in the highest quintile had almost half the odds of developing T2D (OR = 0.50, 95% C.I. 0.32 – 0.77).

**Table 2:** Stepwise approach to examine energy-adjusted zinc as an independent predictor of a new diagnosis of diabetes

	**Quintile of energy-adjusted zinc**					
	**Q1**	**Q2**	**Q3**	**Q4**	**Q5**	**P**
Number of women	1785	1784	1784	1784	1784	
Energy-adjusted zinc [median (min, max)]	−1.25 (-4.8, -0.79)	−0.48 (-0.79, -0.23)	0.01 (-0.23, 0.26)	0.50 (0.26, 0.79)	1.24 (0.79, 4.45)	
Number of diabetics	80	60	59	74	60	
Odds ratio						
• Age adjusted	1.00	0.74 (0.53 to 1.05)	0.73 (0.52 to 1.03)	0.99 (0.67 to 1.28)	0.75 (0.53 to 1.05)	0.319
• Age & non-dietary^†^ factors adjusted	1.00 1.00	0.82 (0.56 to 1.19)	0.65 (0.44 to 0.96)	0.83 (0.58 to 1.19)	0.56 (0.38 to 0.83)	0.010
• Age, non-dietary^†^ and dietary^‡^ factors adjusted	1.00	0.78 (0.53 to 1.15)	0.60 (0.40 to 0.91)	0.77 (0.52 to 1.13)	0.48 (0.31 to 0.75)	0.004
• Age, non-dietary^†^ and dietary^‡^ factors adjusted plus alcohol intake and use of supplements		0.80 (0.54 to 1.17)	0.60 (0.40 to 0.90)	0.78 (0.53 to 1.15)	0.50 (0.32 to 0.77)	0.006

^†^ Non-dietary factors were BMI; smoking status; HRT; exercise group; and history of arthritis, CHD, hypertension, asthma and depression.

^‡^ Dietary factors were energy-adjusted fiber, iron and fat.

Adjustment for family income in the models resulted in a loss of 1300 observations but the p-values for the test for trend were very similar (p = 0.010 for all 3 adjusted models).

The association between the energy-adjusted zinc/iron ratio and odds of developing T2D (Table 3) followed a similar trend to the association observed between total energy-adjusted zinc intake and T2D. After adjusting for age and non-dietary factors a borderline statistically significant association was observed between energy-adjusted zinc to iron ratio and the odds of developing T2D (P_trend_ = 0.073), however there was a consistent decrease in the odds of developing T2D across the quintiles. A statistically significant decrease in the odds of developing T2D across quintiles of energy-adjusted zinc to iron ratio was observed with further adjustment for energy-adjusted dietary factors (p = 0.003) and alcohol intake and use of supplements (p = 0.004). Compared with the lowest quintile of energy-adjusted zinc to iron ratio those in the highest quintile had half the odds of developing T2D (OR = 0.50, 95% C.I 0.30-0.83).

**Table 3:** Stepwise approach to examine zinc/iron ratio as an independent predictor of a new diagnosis of diabetes

	**Quintile of zinc to iron ratio**					
	**Q1**	**Q2**	**Q3**	**Q4**	**Q5**	**P**
Number of women	1785	1784	1784	1784	1784	
Zinc/Iron ratio [median(min, max)]	0.69 (0.28, 0.77)	0.84 (0.77, 0.90)	0.95 (0.90, 1.00)	1.06 (1.00, 1.12)	1.21 (1.12, 1.75)	
Number of diabetics	60	72	71	65	65	
Odds ratio						
• Age adjusted	1.00	1.21 (0.85 to 1.71)	1.19 (0.84 to 1.70)	1.09 (0.76 to 1.56)	1.09 (0.76 to 1.56)	0.885
• Age & non-dietary^†^ factors adjusted	1.00	0.91 (0.61 to 1.33)	0.91 (0.62 to 1.34)	0.73 (0.49 to 1.09)	0.74 (0.50 to 1.10)	0.073
• Age, non-dietary^†^ and dietary^‡^ factors adjusted	1.00	0.76 (0.50 to 1.16)	0.71 (0.46 to 1.11)	0.54 (0.33 to 0.86)	0.50 (0.30 to 0.83)	0.003
• Age, non-dietary^†^ and dietary^‡^ factors adjusted plus alcohol intake and use of supplements	1.00	0.75 (0.50 to 1.14)	0.72 (0.46 to 1.12)	0.54 (0.34 to 0.87)	0.50 (0.30 to 0.83)	0.004

^†^ Non-dietary factors were BMI; smoking status; HRT; exercise group; and history of arthritis, CHD, hypertension, asthma and depression.

^‡^ Dietary factors were energy-adjusted fiber, iron and fat.

## Discussion

In this longitudinal study we observed an inverse association between dietary zinc intake and risk of T2D in a mid-aged female population after adjusting for potential dietary and non-dietary confounders. In addition, we also observed a consistent inverse association between dietary zinc/iron ratio and risk of T2D. This suggests that the proportion of zinc intake in relation to iron is an important determinant of T2D risk. This makes sense given that iron competes with zinc for binding ligands within the intestinal lumen or for transport proteins on the brush border of enterocytes [19]. These findings are consistent with a recent study of 82,297 women (mostly white nurses) aged 30 to 60 years reported by Sun et al. [11], but this is in contrast to the findings of a study conducted by Shi et al [12] in China of 1056 healthy men and women aged 20 years and above. As our study is a representative sample of mid-age, community-dwelling women from across Australia rather than a clinical sample it has high external validity.

Zinc is required for various aspects of cellular homeostasis. It is involved in the catalytic activity of approximately 300 enzymes and plays a role in immune function, cell division, protein and DNA synthesis and apoptosis [2,20]. The human body has no specialised zinc storage system [20] and so humans rely on a daily intake of dietary zinc to maintain health and prevent disease. It is well established that zinc has an insulin-like effect on all insulin sensitive tissues. Insulin exerts its effect by binding the insulin receptor and activating an intracellular signalling cascade mediated by the phosphoinositide 3’-kinase (PI3K)/Akt complex. Zinc (II) ions have been shown to activate this same complex in numerous human cell types [21]. Zinc (II) ions have also been shown to suppress protein tyrosine phosphatases associated with the insulin signalling cascade thus activating the insulin signalling cascade resulting in glucose uptake, increased glycogen synthesis, and decreased gluconeogenesis [21].

The importance of zinc in the euglycaemic state is highlighted by a number of animal studies that have demonstrated that zinc improves hyperglycaemia, glucose intolerance, and insulin resistance [22-24]. A number of molecular and cellular studies have shown that zinc inhibits lipolysis in adipocytes and stimulates glucose uptake in rat and murine adipocytes [24,25]. Further studies have shown that gluconeogenesis was attenuated in rat hepatocytes exposed to zinc and in rat renal cortex slices cultured in the presence of zinc, while glycogen synthesis was stimulated in various cell types exposed to zinc. Moreover, zinc attenuates the high insulin secretory response to glucose in isolated pancreatic islet cell [26]. Considering that zinc is required for the synthesis and release of insulin from pancreatic β cells [27] it follows that low levels of bioavailable zinc may have a significant impact on glucose metabolism and ultimately the risk of T2D.

Studies have documented zinc deficiency in most patients with T2D leading to speculation that zinc may have beneficial effects when administered to diabetic patients [2,7], although the zinc loss may simply reflect increased urinary loss due to diabetic nephropathy. Epidemiological studies have observed that hyperzincuria and hypozincemia are commonly seen in T2D although the mechanism for the urinary loss has not been fully identified [4,5]. Costarelli et al [28] found that subjects with a lower dietary zinc intake display general impairment of their zinc status, an altered lipid profile and increased insulin production in comparison to obese subjects with normal zinc dietary intake. As mentioned above few studies have examined the effect of zinc supplementation in T2D patients with some showing an improvement in glycaemic control. A recent study [29] showed that supplementation either with zinc plus multi vitamin and mineral (MVM) or MVM alone in 96 patients with T2D resulted in a significant favourable effect on blood glucose parameters. This beneficial effect of the zinc and MVM supplements was dependent on the initial fasting blood sugar, indicating that zinc was more effective earlier in the diabetic spectrum and less effective once diabetes was established. A more recent systematic review and meta-analysis of 25 articles, which included 22 studies on T2D, concluded that zinc supplementation has beneficial effects on glycaemic control [30]. The review however had several limitations including differences in zinc doses, sample size, study duration, limited availability of data on zinc intake and variation in baseline parameters and so the conclusions remain in doubt. Despite all this study supporting the effect of zinc in glycaemic control in T2D, there are equally many studies that have found that zinc supplementation have no effect on glycaemic parameters such as Hb1Ac and glucose levels [31-33]. Another study also did not find differences in glycaemic control among patients with T2D treated with oral zinc compare to placebo despite improving zinc status [34]. Hence, despite the positive effects of supplemental zinc observed in animals and cellular studies the connection between dietary zinc supplementation and its role in cellular signalling in humans remains unclear and requires further investigation.

The main strengths of this study are the prospective design, where dietary assessment preceded the development of T2D, and the generalizability, this being a population-based cohort rather than a clinic sample. The main advantage of the prospective design is that it reduces selection bias and potential recall bias. The large sample size also means that it is possible to obtain reasonably stable estimates of incidence rates between successive surveys.

Despite the good generalizability of this study there are some limitations. Though the Pearson correlation coefficient for dietary zinc intake was deemed acceptable because other studies have provided a similar estimate it is still below that observed using other dietary intake methods such as a weighted food record. This is one of the known limitations of using a FFQ to collect dietary information (especially micronutrients) and is due to the lack of homogeneity in food composition tables [35] and over or under reporting of certain foods/food groups. However, the use of FFQs to collect dietary information in large population-based samples is the most cost-effective and feasible method available and the interpretation of findings using an FFQ study needs to consider this limitation. In this study the use of an FFQ to estimate dietary zinc intake will have underestimated the amount of zinc consumed by study participants and biased the effect size towards the null.

Another concern is that the existence of additional environment sources of zinc that may have contributed to the overall intake of zinc. However given the random sample of subjects selected for this investigation this additional exposure is unlikely to have affected some quintiles of zinc intake more than others. Hence, the amount of zinc consumed by all study participants is likely to be an underestimate and this will bias the observed association between dietary zinc and T2D towards the null. Given that an FFQ was used to estimate dietary zinc intake and that there may by some environmental sources of zinc consumption the true estimate of the association will actually be larger than that observed in this study.

There is also the possibility that other confounding variables may not have been controlled for in the analysis and this suggests that our estimate of the effect of zinc on T2D risk may be subject to some residual confounding; however given that the most important dietary (including multivitamin use) and non-dietary confounders have been controlled for this residual confounding is likely to be small.

A further limitation is that dietary assessment was carried out at one time only in survey 3. However, the dietary habits of the participants are unlikely to have changed significantly over the 6-year period from survey 3 to survey 5, as they are mid-aged and dietary patterns are likely to remain stable. Instead of excluding participants who develop comorbidities during the follow-up period we controlled for these in the analysis. This was done to account for any change in dietary habit that may have occurred following the development of comorbid conditions. Although adjustments were made for all known potential confounders, residual confounding cannot be entirely excluded.

This study also considered the use of supplementary zinc as and additional source and though the quantity of zinc in the supplements was not known, analysis showed that the proportion of people taking supplements were not significantly different between quintiles of zinc intake. Although multiple dietary assessments are useful to reduce random measurement error, given that single dietary assessment is likely to bias results toward the null the fact that we still observed a significant association is an indication that the association between dietary zinc and dietary zinc to iron ratio is likely to be robust and even stronger than that observed in this study.

Although T2D was self-reported we do not consider this a significant limitation as the kappa statistic for agreement between self-report of diabetes and New South Wales Admitted Patient Data Collection (APDC) hospital records in the mid-aged cohort was 0.75 while the prevalence adjusted figure is 0.93 (unpublished data).

## Conclusions

In conclusion, the current study confirms the recent findings by Sun et al [11] that higher total dietary zinc intake and high zinc/iron ratio is associated with lower risk of T2D in women. Future research should examine this association in both men and women across different age groups. A positive finding in future studies should prompt further research to determine if zinc supplementation may reduce the risk of developing T2D.

## Abbreviations

ALSWH: Australian longitudinal study on women’s health; DQES: Dietary questionnaire for epidemiological studies; FFQ: Food frequency Questionnaire; HRT: Hormone replacement therapy; HT: Hypertension; MVM: Multivitamin and minerals; S1: Survey 1; S2: Survey 2; S3: Survey 3; S4: Survey 4; S5: Survey 5; T2D: Type 2 diabetes.

## Competing interests

The authors declare that they have no competing interests.

## Authors’ contributions

KV: Conceptualization of the idea, study design, literature search, data interpretation and development of manuscript. MMcE: Conceptualization of the idea, study design, literature search, data interpretation and development of manuscript. AM: Conceptualization of the idea, study design and manuscript preparation. MRI: Conceptualization of the idea, study design and manuscript preparation. DS: Data analysis, data interpretation and manuscript preparation. AP: Original concept, design of the analysis and manuscript preparation. JB: Data collection and development of manuscript. DL: Data collection and development of manuscript. JA: Conceptualization of the idea and development of manuscript. ZS: Development of manuscript. All authors read and approved the final manuscript.

## Pre-publication history

The pre-publication history for this paper can be accessed here:

http://www.biomedcentral.com/1472-6823/13/40/prepub
